# Let-7d suppresses growth, metastasis, and tumor macrophage infiltration in renal cell carcinoma by targeting COL3A1 and CCL7

**DOI:** 10.1186/1476-4598-13-206

**Published:** 2014-09-06

**Authors:** Boxing Su, Wei Zhao, Bentao Shi, Zhongyuan Zhang, Xi Yu, Feng Xie, Zhongqiang Guo, Xiaoyu Zhang, Jin Liu, Qi Shen, Jinghua Wang, Xuesong Li, Zhiqian Zhang, Liqun Zhou

**Affiliations:** Department of Urology, Peking University First Hospital & the Institute of Urology, Peking University, Beijing, 100034 China; National Urological Cancer Center, Beijing, 100034 China; Department of Cell Biology, Peking University School of Oncology, Beijing Cancer Hospital and Institute, Beijing, 100142 China; Department of Urology, Peking University Shenzhen Hospital, Shenzhen, Guangdong 518036 China; Department of Urological pathology, Peking University First Hospital & the Institute of Urology, Peking University, Beijing, 100034 China

**Keywords:** Renal cell carcinoma, MicroRNA, Let-7

## Abstract

**Background:**

MicroRNAs are endogenous small noncoding RNAs that are functionally involved in numerous critical cellular processes including tumorigenesis. Data mining using a microRNA array database suggested that let-7d microRNA may be associated with renal cell carcinoma (RCC) malignant progression. Here, we performed further analyses to determine whether let-7d is functionally linked to RCC malignancy.

**Methods:**

Quantitative real-time PCR was used to determine the level of mature let-7d in RCC clinical specimens and its correlation with clinicopathological data. Immunohistochemical staining was conducted to characterize the stroma of RCC. Let-7d overexpressing RCC cell lines combined with mouse models bearing cell-derived xenografts and patient-derived xenografts were used to assess the functional role of let-7d *in vitro* and *in vivo*.

**Results:**

Downregulation of let-7d in clinical RCC samples was associated with advanced tumor grade and T stage and increased vascular invasion. An inverse relationship between let-7d expression and macrophage infiltration was found in clinical RCC samples. Functional studies indicated that ectopic expression of let-7d significantly inhibited RCC cell proliferation, migration, and peripheral blood monocyte (PBMC) recruitment *in vitro*, as well as tumor growth, metastasis, and tumor macrophage infiltration *in vivo. In silico* analysis and subsequent experimental validation confirmed collagen, type III, alpha 1 (COL3A1) and C-C subfamily chemokine member CCL7 as direct let-7d target genes. The addition of COL3A1 and CCL7 counteracted the inhibitory effects of let-7d on RCC cell proliferation, migration, and PBMC recruitment. The inhibition of let-7d increased cell proliferation, migration, and PBMC recruitment by the enhanced expression of COL3A1 and CCL7 genes *in vitro*. The mRNA levels of COL3A1 and CCL7 were inversely correlated with let-7d level in RCC clinical specimens.

**Conclusions:**

These results suggest that let-7d may suppress RCC growth, metastasis, and tumor macrophage infiltration at least partially through targeting COL3A1 and CCL7.

**Electronic supplementary material:**

The online version of this article (doi:10.1186/1476-4598-13-206) contains supplementary material, which is available to authorized users.

## Background

Renal cell carcinoma (RCC) is one of the common urological cancers usually with poor prognosis [1]. RCC accounts for approximately 3% of adult malignancies and for approximately 90–95% of neoplasms arising from the kidney [2, 3]. Surgical treatment can cure 60–70% of localized RCC but only prolongs survival in most metastatic RCC patients [4]. RCC is relatively resistant to radiation and chemotherapy [5]. Considerable progress has been made in the therapy of patients with localized RCC; however, the treatment options for patients with metastatic RCC are very limited [6]. Therefore, identification of new biomarker and anti-tumor agents, particularly with better efficiency against metastatic RCC, remains a high priority.

MicroRNAs (miRNAs) are a large gene family of short (21–23 nucleotides) non-coding RNAs. The single-stranded miRNA binds through imperfect base pairing with the 3′ untranslated region (3′-UTR) of target mRNAs and causes either repression of translation or degradation of mRNAs [7]. Each of the hundreds of known miRNAs can epigenetically downregulate many target genes that participate in various biological processes including cell proliferation, apoptosis, migration, differentiation and development [8].

It is increasingly apparent that the interplay between cancer cells and their stroma is of great importance to tumorigenesis and progression [9]. Tumor-associated stroma is composed of multiple stromal cell types, such as cancer-associated fibroblasts, immune inflammatory cells, and endothelial cells, as well as a variety of extracellular matrix (ECM) proteins, such as fibronectin and collagen. The tumor-associated stroma constitutes an important compartment of tumor microenvironment, which can enable primary, invasive, and then metastatic growth of tumor through crosstalk with cancer cells [10]. Recent studies have shown that the regulatory role of miRNAs during cancer progression is not limited to cancer cells, and that miRNAs are also involved in the activation and transition of tumor stromal cells [11]. Thus, miRNAs have emerged as a potent regulator in the crosstalk between cancer and stromal cells in the tumor microenvironment.

The let-7 family, originally identified in *Caenorhabditis elegans,* consists of 13 family members that are highly conserved across the animal phylogeny from *C. elegans* to human. Nine members of the let-7 family have been identified in humans [12]. Let-7 functions as a heterochronic gene in many species. It is undetectable in human and mouse embryonic stem cells, but increases during embryogenesis and differentiation [13]. High let-7 expression levels are subsequently maintained in a variety of adult tissues [14]. Conversely, let-7 is frequently downregulated in many human malignancies, such as lung cancer, breast cancer, and hepatocellular carcinoma [15–17], possibly reflecting the reverse embryogenesis process that occurs during oncogenesis [18].

Recently, we have identified a subset of miRNAs that are low expressed in RCC relative to adjacent normal tissues by using microarray (unpublished data). The current study was designed to explore the function of one of these miRNAs, let-7d, in RCC progression.

## Results

### Let-7d is downregulated in human RCC cell lines and clinical RCC samples

**Figure 1:**
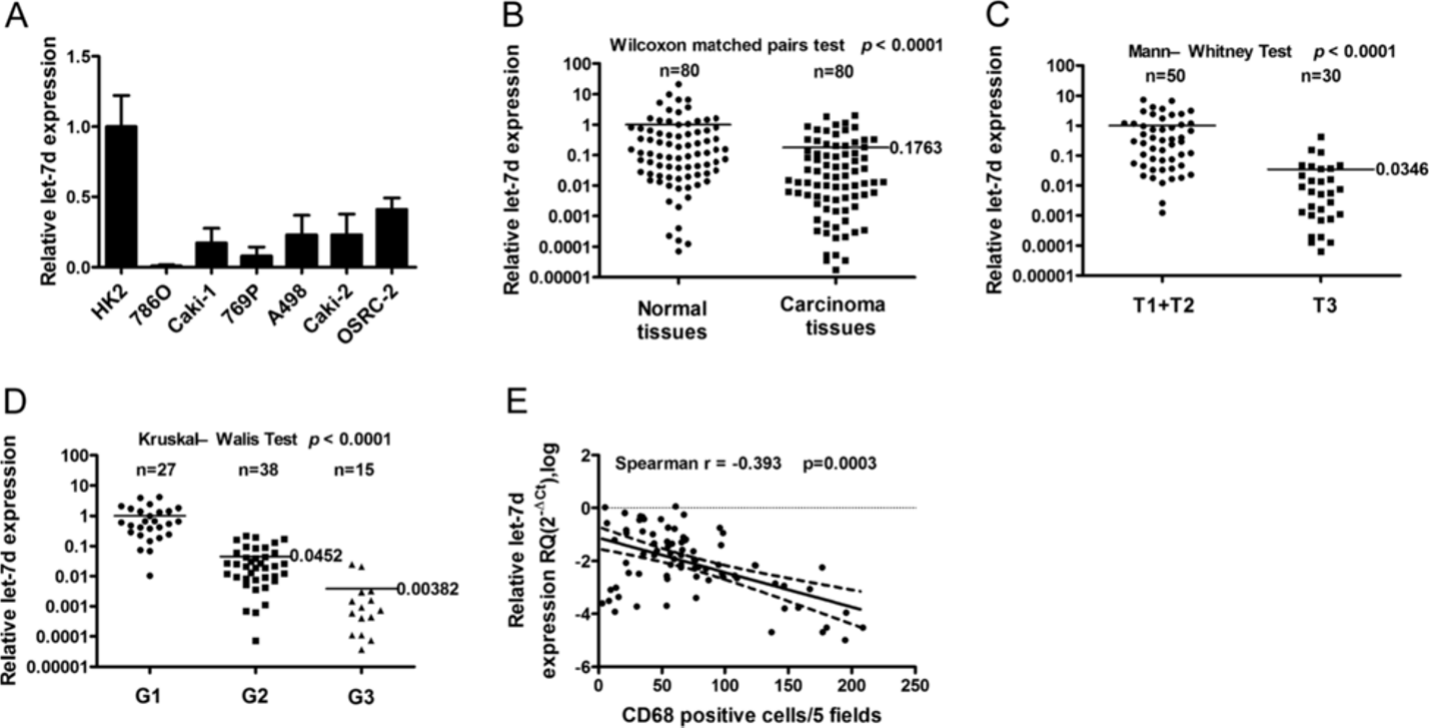
**Let-7d expression in RCC cell line and tumor samples, and correlation with tumor stromal cells. (A)** Real-time RT-PCR analysis of relative let-7d expression in human cell lines. The data represent the average ± SD of three independent RT-PCR results. **(B)** The relative expression of let-7d in 80 RCC tissues and matched adjacent normal tissues were analyzed by real-time RT-PCR. **(C, D)** Real-time RT-PCR analysis of relative let-7d expression in 80 RCC tissues in different T stages **(C)** and tumor grades **(D)**. Horizontal lines in **(B–D)** represent the mean values of relative let-7d expression for each series of samples. Note that **(B–D)** were generated from the same data as Table 1. **(E)** A linear regression and correlation among relative let-7d expression in a log scale vs. CD68 positive cell counts in five independent fields under × 20 objective lens is shown with r (Spearman) and P-values indicated.

We examined let-7d expression in several human RCC cell lines by quantitative real-time RT-PCR. The normal renal tubule epithelial cell line HK-2 had significantly higher let-7d level than the RCC cell lines (Figure 1A). We then examined let-7d expression in 80 clinical RCC samples and their matched adjacent tissues. The mean let-7d level in RCC was 17.6% of that in the matched adjacent tissues (Figure 1B). Given that let-7 family members are sometimes indistinguishable [19], we also checked the expression of let-7a, the only let-7 family member that was reported to function as a tumor suppressor in RCC cell lines [20]. As shown in Additional file 1: Figure S1 (Supplementary Data), no difference of let-7a expression was found between tumor tissues and the matched adjacent tissues. These data indicate that the decrease of let-7d expression in RCC may be specific.

### Decreased let-7d expression is associated with advanced T stages and tumor grades in RCC patients

A total of 80 RCC patients were analyzed for the correlation between clinicopathological characteristics and the let-7d level in RCC (Table 1, Figures 1C and 1D). There was no significant correlation between let-7d level and age at surgery or histological subtype. Let-7d level in RCC was significantly lower in male patients than in female patients (Table 1), but the difference between males and females was not found in the adjacent normal tissues (Additional file 1: Figure S2). Let-7d expression was significantly lower in T3 stage RCC than in T1 and T2 stage RCC, and decreased remarkably with advanced degree of RCC differentiation. Moreover, let-7d expression in RCC with vascular invasion was significantly lower than that in RCC without vascular invasion. Therefore, downregulation of let-7d is highly correlated with the malignant degree of RCC.

**Table 1:** **Relationship between let-7d expression and clinicopathological features in RCC patients**

		Let-7d expression ^1^(RQ: 2 ^-ΔCt^)		
Variable	Case no.	Median	Range	P ^2^
Gender				0.036
Male	51	5.7 × 10^-3^	1.0 × 10^-5^-1.06	
Female	29	0.027	3.1 × 10^-5^-1.14	
Age				0.19
<60(median)	35	7.4 × 10^-3^	2.1 × 10^-5^-0.655	
≥60	45	8.5 × 10^-3^	1.0 × 10^-5^-1.14	
T stage				<0.0001
T1 and T2	50	0.046	1.9 × 10^-4^-1.14	
T3	30	9.2 × 10^-4^	1.0 × 10^-5^-0.067	
G grade				<0.0001
G1	27	0.28	2.8 × 10^-3^-1.14	
G2	38	5.8 × 10^-3^	2.1 × 10^-5^- 0.061	
G3	15	2 × 10^-4^	1.0 × 10^-5^-6.8 × 10^-3^	
Histological type				0.069
papillary RCC	2	0.026		
chromophobe RCC	3	2 × 10^-4^		
clear cell RCC	75	0.011	1 × 10^-5^-1.14	
Vascular invasion				0.010
positive	15	0.0014	1 × 10^-5^- 0.18	
negative	65	0.017	2.1 × 10^-5^-1.14	

^1^Let-7d expression was normalized to U6.

^2^Mann–Whitney test for the comparison between two groups or Kruskal-Walis test for more groups.

### Let-7d expression is inversely correlated with tumor macrophage infiltration

We investigated the association between let-7d expression and changes of several tumor stromal cells including macrophages, cancer-associated fibroblasts, T-regulatory cells (Tregs), and mast cells, which have been well documented to associate with tumor progression [10, 21, 22]. Representative micrographs of the above-mentioned stromal cells are shown in Additional file 1: Figure S3. Statistics of positive cell counts and their correlation with let-7d expression level in tumor tissue are shown in Additional file 2: Table S2. There were no statistically significant links between let-7d level and counts of cancer associated fibroblasts, Tregs, and mast cells, but an inverse correlation between let-7d and macrophage number was observed (Spearman’s r = -0.393, *P* = 0.0003) (Figure 1E). This suggests a regulatory role of let-7d for macrophage infiltration in RCC stroma.

### Overexpression of let-7d impedes RCC cell growth and migration of RCC cells *in vitro*

**Figure 2:**
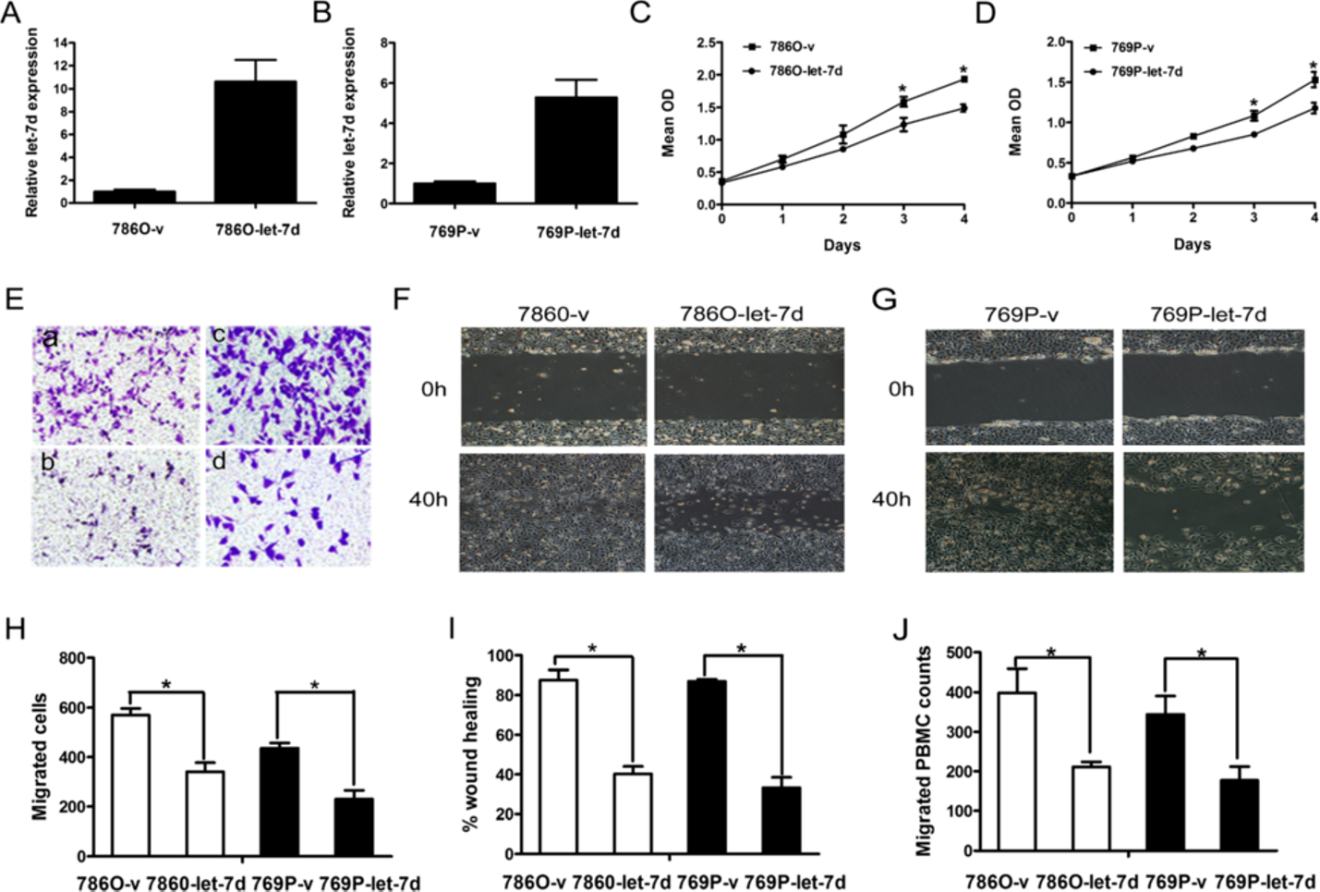
**Ectopic expression of let-7d** ***in vitro*** **. (A, B)** Relative expression of let-7d in pri-let-7d lentivirus-infected cells (786O-let-7d or 769P-let-7d) compared with vehicle control vector-infected cells (786O-v or 769P-v). **(C, D)** Cell proliferation was evaluated using CCK-8. **(E)** Representative images of migrated cells evaluated by Boyden chamber assay. (a) 786O-v, (b) 786O-let-7d, (c) 769P-v, (d) 769P-let-7d cells. Original magnification: ×100. **(F, G)** Representative images of wound gaps in let-7d overexpression and control cells at different time points. Original magnification: ×40. **(H, I)** The quantification results of migrated cells and percent wound healing are represented as the mean ± SD of three independent experiments with five random fields counted for each chamber and wound area. **(J)** PBMC chemotaxis was evaluated by chemotaxis assay. Results are expressed as mean ± SD of three independent experiments. *P < 0.05.

The low let-7d level in RCC prompted us to investigate whether let-7d functions as a tumor suppressor in RCC. We infected the RCC cell lines 786O and 769P with pri-let-7d lentivirus and measured its effects on cell proliferation and migration *in vitro*. After the infection, let-7d levels in 786O and 769P cells were upregulated (9.6- and 5.3-fold, respectively; Figures 2A and 2B), and their proliferation and migration evaluated by proliferation assay (Figures 2C and 2D), Boyden chamber assay (Figures 2E and 2H), and wound-healing assay (Figures 2F, G and I) were significantly inhibited, as compared with the cells infected with the control lentivirus. These results indicate that let-7d may be a tumor suppressor in RCC.

### Overexpression of let-7d decreases the monocyte recruitment of RCC cells

To investigate the potential mechanism of the inverse correlation between let-7d expression and tumor macrophage infiltration in RCC, we performed chemotaxis assays with PBMCs in the upper chamber and conditioned medium from let-7d-overexpressing 786O or 769P cells or from control cells in the lower chamber. We found that overexpression of let-7d in RCC cells led to decreased migration of PBMCs compared with vehicle control (Figure 2J). These results suggest the regulatory role of let-7d in the inhibition of chemotactic activity leading to the decrease of macrophage infiltration in RCC.

### Overexpression of let-7d inhibits the growth, metastasis, and tumor macrophage infiltration of RCC in animal models

**Figure 3:**
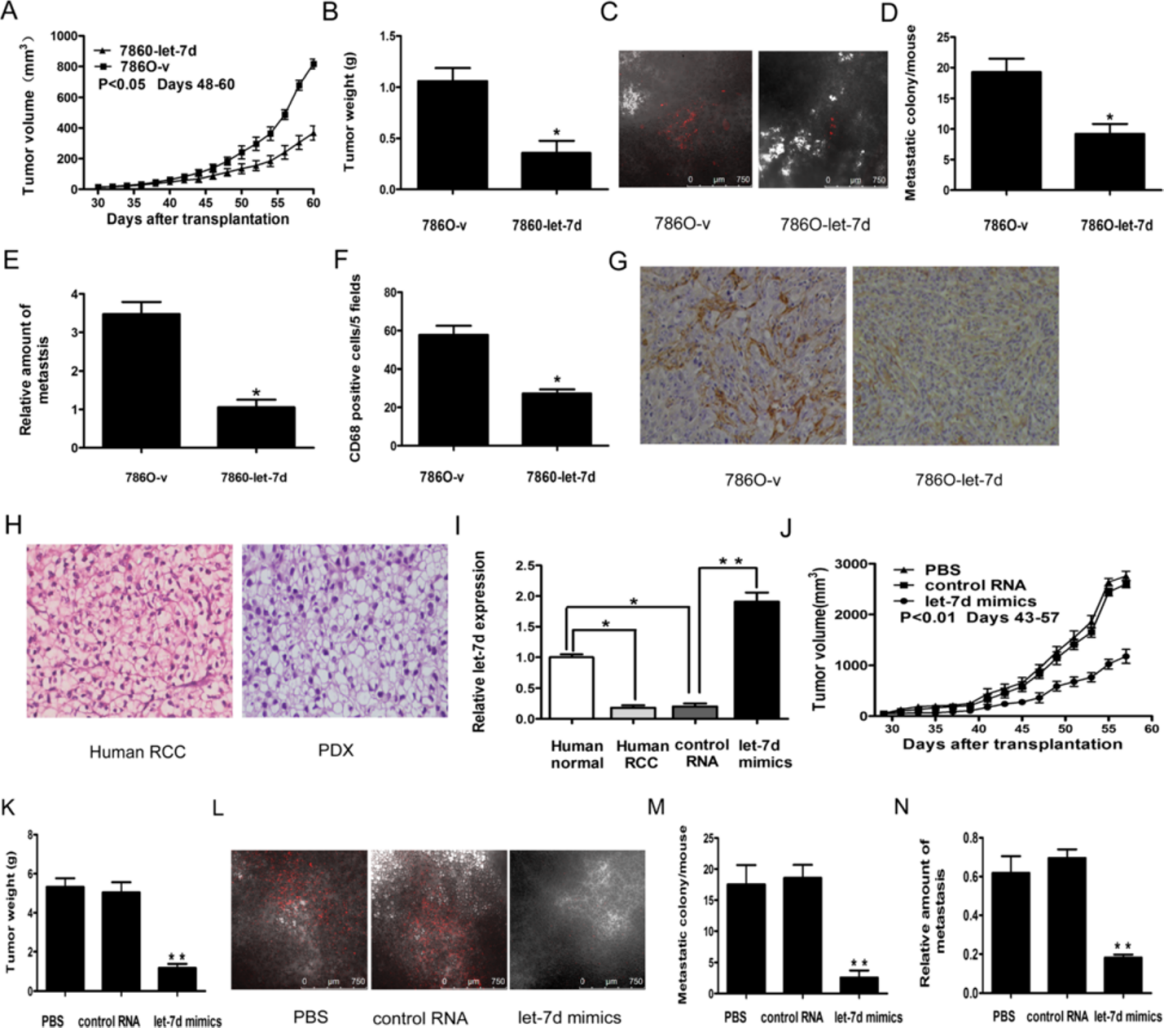
**Overexpression of let-7d** ***in vivo.*** **(A, B)** Growth curves and average net weights of xenografts formed by 786O-v (n = 5) and 786O-let-7d cells (n = 5). **(C, D)** Representative micrographs and quantitative data of metastatic colonies in mice lung. DiI-positive lung metastatic colonies were photographed and counted under a laser confocal microscope. **(E)** Real-time PCR quantification of relative Alu-sequence expression in mice lung. **(F)** Quantitative data of the mean CD68 positive cells per five fields in each group. **(G)** Representative picture of IHC staining of CD68 positive cells. Original magnification: ×200. Data in **(A–F)** represent the mean ± SD of five mice per group. *P* values were obtained by the two-tailed Student’s t-test. *P < 0.05. **(H)** HE staining of the patient tumor and its corresponding derived xenograft (2nd passage). Original magnification: ×200. **(I)** Real-time RT-PCR analysis of let-7d expression in human RCC tissue, paired normal adjacent tissue, and corresponding PDX tumor tissues 1 week after intratumoral injection of let-7d mimics or control RNA. **(J, K)** Growth curves and average net weights of xenografts injected with let-7d mimics (n = 7), control RNA (n = 7) or PBS control (n = 7). **(L, M)** Representative pictures and quantitative data of metastatic colonies in mice lung. **(N)** Real-time PCR quantification of relative human-specific Alu-sequence expression in mice lung. The values in **(J–N)** are presented as the mean ± SD of seven mice. *P* values were obtained by one-way ANOVA. **P < 0.01 (let-7d mimics vs. PBS or control RNA).

We then used the cell-derived xenograft (CDX) model in nude mice to investigate the effects of let-7d overexpression on RCC. The growth of let-7d overexpressing 786O cells was significantly inhibited and the mean tumor weight was reduced by 66.4% as compared with the controls (Figures 3A and 3B). The number of metastatic colonies (Figures 3C and 3D) and the quantification of human-specific Alu-sequence (Figure 3E) in the mouse lung were also significantly reduced. Tumor macrophages as indicated by CD68^+^ cells in let-7d overexpressing xenografts were decreased by 52.7% as compared with the controls (Figures 3F and 3G).

We further used the patient-derived xenograft model (PDX), a valuable tool for preclinical trials, in NOD/SCID mice to evaluate the effect of let-7d overexpression on RCC growth and metastasis. Early passage (2nd passage) of the RCC xenografts recapitulated the morphologic features of the original clinical tumor (Figure 3H). The let-7d level in PDX was similar to that in original human RCC and was significantly decreased as compared with that in normal adjacent tissues (Figure 3I). By intratumoral injection of cholesterol-conjugated let-7d mimics [23], we found that let-7d level in tumors was elevated by 8.6-fold as compared with that in controls (Figure 3I). Consistent with the results in the CDX model, tumor growth was suppressed and tumor weight was decreased after the intratumoral injection of let-7d mimics (Figures 3J and 3 K). The number of metastatic colonies and the quantification of human-specific Alu-sequence in mouse lung were also reduced (Figures 3L–N). The results from both the CDX and PDX animal models suggest that overexpression of let-7d in RCC results in dramatic repression of RCC growth, metastasis, and macrophage infiltration, and that administration of let-7d to tumor tissue may be a therapeutic alternative for RCC.

### COL3A1 and CCL7 are direct let-7d target genes in RCC cells

**Figure 4:**
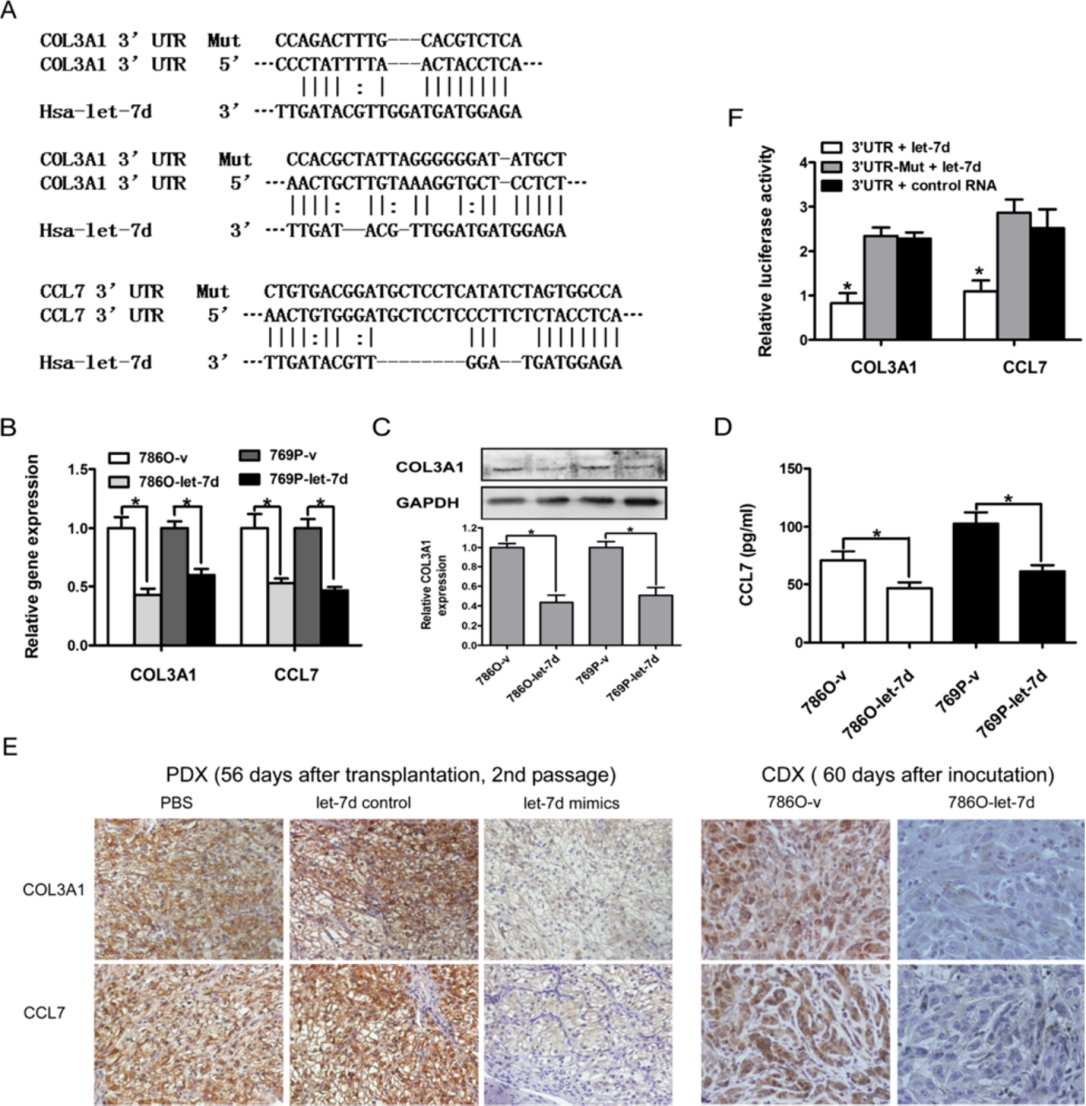
**Let-7d directly targets COL3A1 and CCL7 in RCC cells. (A)** Sequence alignment of human let-7d seed sequence within the 3′-UTRs of COL3A1 and CCL7. The mutated sequence in the putative let-7d binding sites for each gene is shown in the top of each gene set. **(B)** Real-time RT-PCR analysis of COL3A1 and CCL7 expression in RCC cells. **(C, D)** The protein levels of COL3A1 and CCL7 in RCC cells were detected by western blot and ELISA. Corresponding densitometry of each band is presented in a bar graph. Data presented in **(B–D)** are the mean ± SD of three independent experiments. *P < 0.05. **(E)** IHC staining of COL3A and CCL7 expression in the tumor sections from each experimental group of PDX and CDX model. Original magnification: ×200. **(F)** Luciferase activity of various reporter plasmids. Statistical significance was obtained using one way ANOVA. The data represent the mean ± SD of three independent experiments with triplicates of each sample.*P < 0.05.

To investigate the mechanism involved in the suppression effect of let-7d on tumor growth, metastasis, and macrophage infiltration in RCC, we first performed *in silico* search for the target mRNAs using three algorithms (MiRanda, PicTar, and TargetScan), and obtained a list of predicted target mRNAs of let-7d. The genes potentially involved in tumor growth, metastasis, and chemotaxis activity of RCC were then selected through data mining using the Gene Expression Omnibus Database [24]. Preliminary semi-quantitative RT-PCR screening identified downregulation of COL3A1 and CCL7 mRNA following forced expression of let-7d (data not shown). Quantitative real-time RT-PCR, western blot and ELISA confirmed that the expressions of COL3A1 and CCL7 were significantly decreased at both mRNA and protein levels in 786O and 769P cells transfected with pri-let-7d (Figures 4B–D). Immunohistochemistry staining also showed that COL3A1 and CCL7 were decreased in CDX and PDX samples in which let-7d was overexpressed (Figure 4E).

To determine whether the two mRNAs are *bona fide* targets of let-7d, the 3’-UTRs flanking the putative binding sites of let-7d in COL3A1 and CCL7 mRNAs were cloned into reporter plasmids immediately downstream of luciferase cDNA. The reporter plasmids containing the mutant putative binding sites of let-7d were also constructed as the controls (Figure 4A). Luciferase activities decreased significantly in the cells transfected with the wild-type reporter plasmids but not in those transfected with the mutant plasmids (Figure 4F). These findings indicate that the 3’-UTRs in COL3A1 and CCL7 mRNAs are the target sites, through which let-7d modulates the expressions of COL3A1 and CCL7.

### Rescue of COL3A1 and CCL7 overcomes the effects of let-7d

**Figure 5:**
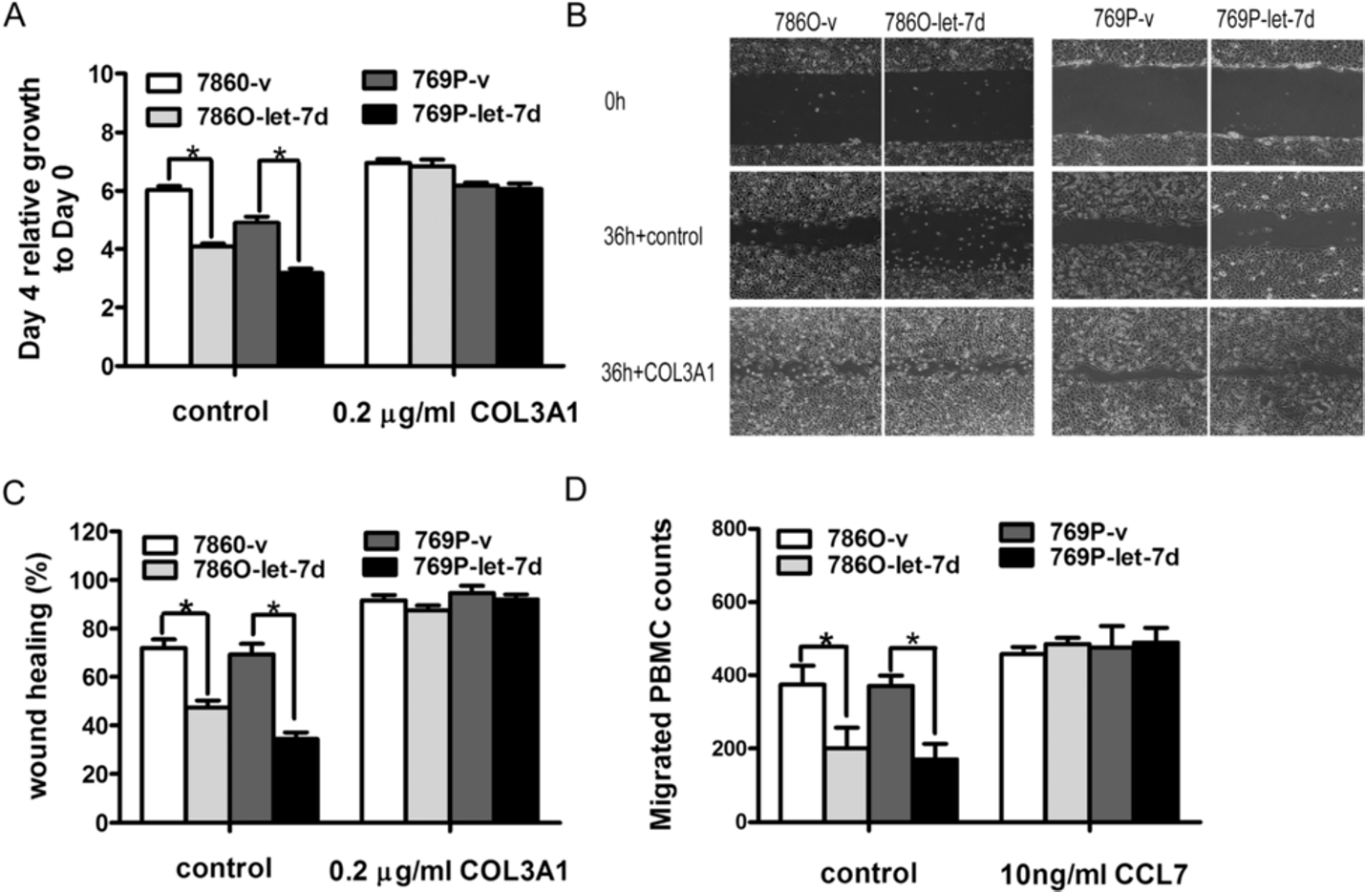
**Rescue of COL3A1 and CCL7 in RCC cells. (A)** Proliferation assay was performed with or without the addition of 0.2 μg/mL type III collagen by CCK-8. **(B)** Representative images of the wound gaps in cells cultured with or without 0.2 μg/mL type III collagen at indicated time point. Original magnification: ×40. **(C)** Percent wound healing is shown as the mean ± SD of the three experiments. **(D)** Migrated PBMC cells were evaluated by chemotaxis assay with or without 10 ng/mL CCL7. Results are expressed as mean ± SD of three independent experiments. *P < 0.05.

To confirm that COL3A1 and CCL7 functions downstream of let-7d, we performed rescue experiments by culturing RCC cells in the presence of purified COL3A1 or CCL7. Addition of 0.2 μg/mL COL3A1 restored the growth and migration of 786O-let-7d and 769P-let-7d cells (Figure 5A-C). Addition of 10 ng/mL CCL7 restored the macrophage recruitment of these two cells (Figure 5D). However, addition of COL3A1 did not reverse the inhibitory effects of let-7d overexpression on macrophage recruitment, and CCL7 did not impact growth and migration of RCC cells (data not shown). These data demonstrate that let-7d suppresses RCC cell growth, metastasis, and macrophage recruitment by directly targeting COL3A1 and CCL7.

### Inhibition of let-7d in OS-RC-2 cells increased the expression of COL3A1 and CCL7 and enhanced cell proliferation, migration, and monocyte recruitment of the cells

**Figure 6:**
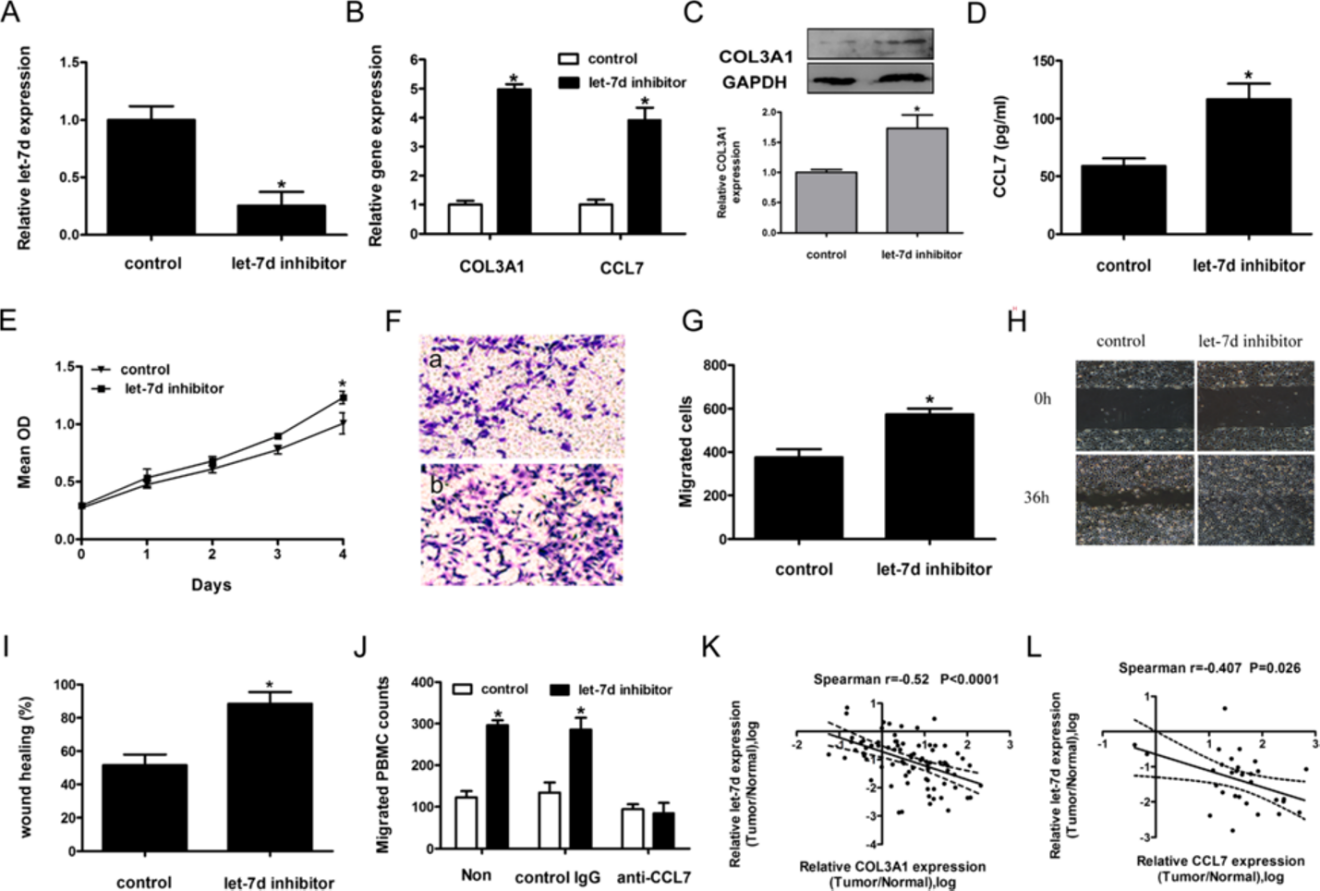
**Inhibition of let-7d in OS-RC-2 cells. (A, B)** Real-time RT-PCR quantification of relative expression of let-7d **(A)**, COL3A1 and CCL7 **(B)** in let-7d inhibitor or negative control transfected OS-RC-2 cells. **(C)** Western blot and **(D)** ELISA of COL3A1 and CCL7 expression. Corresponding densitometry of each band is presented in a bar graph. Data presented are the mean ± SD of three independent experiments. **(E)** Proliferation assay was performed by CCK-8. **(F, G)** Representative images and quantitative data of migrated negative control- (a) or let-7d inhibitor- (b) transfected OS-RC-2 cells evaluated by Boyden chamber assay. Original magnification: ×100. **(H)** Representative images of wound gaps in let-7d inhibitor- and control-transfected OS-RC-2 cells at different time points. Original magnification: ×40. **(I)** The quantification results of percent wound healing are represented as the mean ± SD of three independent experiments. **(J)** PBMC chemotaxis was evaluated by chemotaxis assay with the presence of 1 μg/mL CCL7 neutralizing antibody or control IgG in the conditioned medium. Results are expressed as mean ± SD of three independent experiments. *P < 0.05. **(K, L)** The linear regression and correlation between let-7d and COL3A1 mRNA levels in all 80 RCC tissues **(K)** and between let-7d and CCL7 mRNA levels in all 30 T3 stage RCC tissues from the same set **(L)**. Expression status is shown as the tumor/non-tumor ratio in a log scale.

To further assess whether let-7d is involved in the proliferation, migration, and tumor macrophage infiltration of RCC by targeting COL3A1 and CCL7, we inhibited the expression of let-7d with a synthesized let-7d inhibitor in a relatively low metastatic OS-RC-2 cell line with high endogenous let-7d expression level (Figure 6A). The levels of endogenous COL3A1 and CCL7 mRNA were increased by 3.1- and 2.9-fold, respectively, upon let-7d inhibition compared with the negative control (Figure 6B). Furthermore, western blot and ELISA assay showed that the protein levels of these two genes were upregulated following let-7d repression (Figure 6C, D). The inhibition of let-7d in OS-RC-2 cells significantly increased cell proliferation, migration, and PBMC chemotaxis. Additionally, the increased chemotaxis was eliminated by the addition of 1 μg/mL CCL7 neutralizing antibody in conditioned medium (Figure 6E–J). These data further support the notion that inhibition of let-7d in RCC increases cell proliferation, migration, and macrophage recruitment through the modulation of its targets COL3A1 and CCL7.

### Let-7d expression inversely correlated with COL3A1 and CCL7 mRNA levels in RCC tissues

We analyzed the relationship between level of let-7d and the mRNA levels of COL3A1 and CCL7 in clinical RCC tissues by quantitative real-time RT-PCR Significant inverse correlation was found between let-7d level and COL3A1 mRNA level in the 80 clinical RCC samples (Figures 6K and 6 L), and between let-7d level and CCL7 mRNA level in the clinical RCC samples with T3 stage.

## Discussion

Let-7d belongs to the let-7 family that functions as tumor suppressor in many types of cancer [12]. Reported direct targets of let-7 include oncogenes such as RAS, MYC, and HMGA2 [25]. In this study, we demonstrated the tumor suppressive role of let-7d in RCC and validated that the targets of let-7d were COL3A1, an important stroma component, and CCL7, a chemokine attracting monocytes to tumor tissue. Let-7d expression was negatively correlated with COL3A1, CCL7 and CD68^+^ cells in RCC tissues.

Collagen is the most abundant ECM protein in stroma, and contributes to the tumor progression in tumor stroma [10]. Elevated deposition of collagen has been particularly associated with an altered stroma during breast tumorigenesis and correlated with increased breast cancer risk [26]. Lysyl oxidase is an ECM crosslinking enzyme. Collagen crosslinking-mediated matrix stiffening increases integrin clustering, which leads to phosphorylation of focal adhesion kinase and activation of extracellular signal-regulated kinase [27]. These changes are all involved in cell migration, invasion and proliferation, leading to tumor progression [28]. COL3A1, also known as collagen, type III, alpha 1, is a fibrillar collagen found in extensible connective tissues. The increase of COL3A1 and COL1A1 are found in epithelial ovarian cancers and are prognostic markers of poor prognosis [29]. Interestingly, COL3A1 is the target of miR-29 family, and downregulation of this miRNA family is responsible for the increased invasiveness of lung cancer [30, 31]. Here, we validated through *in vitro* and *in vivo* research that COL3A1 is a functional direct target gene of let-7d. Importantly, COL3A1 expression was inversely correlated with let-7d levels in RCC clinical specimens. Our findings suggest the involvement of the let-7d-COL3A1 regulatory pathway in RCC growth and metastasis.

Macrophages, which are abundant in the tumor microenvironment, were proven to promote cancer initiation and malignant progression by persuasive clinical and experimental evidence [32]. Tumor-associated macrophages are also involved in tumor progression of RCC and can be used as a potential therapeutic target for metastatic RCC [33]. CCL7 (monocyte chemotactic protein-3, MCP-3) is a member of the C-C chemokine subfamily. CCL7 has been found to be overexpressed in gastric cancer tissues and is associated with tumor lymph node metastasis and poor prognosis [34]. Furthermore, CCL7 is more abundant in metastatic tumor site than in the primary site, and is associated with macrophage infiltration in tumor [35]. For example, CCL7 is higher in brain metastatic RCC than in primary RCC [36]. CCL2 is another potent macrophage chemoattractant chemokine belonging to the same chemokine family with CCL7. CCL2 recruits macrophages to facilitate metastasis of breast cancer [37]. In RCC [38], however, CCL2 was shown to be of minor importance in the recruitment of macrophages that preserve diverse tumor-promoting functions. CCL7 promotes the invasion and migration of oral squamous cell carcinoma cells through directly binding to its receptor [39]. Interestingly, we found that the suppressed proliferation and migration in let-7d overexpressing RCC cells could not be restored by the addition of exogenous CCL7 alone, probably due to the multiple pathways downstream of let-7d or the lack of functional CCL7 receptor in RCC cells. However, a strong inverse correlation between let-7d expression and number of infiltrated macrophage was found in our clinical RCC samples. Therefore, there is an indirect role of CCL7 in RCC malignancy via the let-7d-CCL7-macrophage chain. The negative correlation between let-7d expression and CCL7 in T3 stage RCC tissues additionally highlights the role of CCL7 in tumor invasion and metastasis. CCL7 may be functionally involved in RCC malignant progression and may be used in chemokine target therapy for RCC.

In summary, our results indicate that the tumor suppression role of let-7d in RCC may be partially ascribed to its ability to decrease collagen expression and macrophage recruitment through targeting COL3A1 and CCL7 mRNAs. If sufficient let-7d is present in RCC, tumor stroma will be remodeled and cancer cells will be suppressed. It should be noted that let-7d may possess various functions owing to its pleiotropic regulation of genes. It is our expectation that more functional let-7d target genes will be identified in the near future. Further studies are required to fully illustrate their functional roles and interactions with tumor-associated stroma and to determine whether let-7d can be used for the therapy of metastatic RCC, as described in other cancers [23].

## Conclusions

This study demonstrated that let-7d may suppress RCC growth, metastasis and tumor macrophage infiltration at least partially through targeting COL3A1 and CCL7. Our findings suggest let-7d as a promising target for metastatic RCC therapies.

## Methods

### Clinical samples and cell lines

A total of 80 paired RCC tissues and adjacent normal tissues were obtained with informed consent from patients who underwent surgical resection at Peking University First Hospital between 2012 and 2013. The study was approved by Review Board of Peking University First Hospital. All histological subtypes were classified by the Heidelberg classification [40]. Pathological T stage was classified according to the 2010 TNM classification system [41]. Tumor grade was assessed according to Fuhrman nuclear grade [42]. The presence of vascular invasion includes either microvascular invasion, renal vein invasion or inferior vena cava invasion. Immortalized renal proximal tubule epithelial cell line of HK-2 and human RCC cell lines of A498, 769P, 786O, Caki-1 and Caki-2 were purchased from ATCC (MA, USA). Human RCC cell line OS-RC-2 was obtained from the cell bank at Chinese academy of sciences. Transformed human embryonic kidney cells 293FT were purchased from Invitrogen. A498, 769P, 786O, and OS-RC-2 cells were maintained in RPMI 1640 medium, HK-2, Caki-1 and Caki-2 cells in DMEM/F12 (50:50, v/v) medium, and 293FT cells in DMEM. These media were supplemented with 10% fetal bovine serum (HyClone), and penicillin (100 U/mL)/streptomycin (100 μg/mL) (Invitrogen).

### Peripheral blood mononuclear cell (PBMC) isolation

Heparinized venous blood obtained from RCC patients with informed consent was diluted 1:5 with phosphate buffer saline (PBS) and the 40 mL diluted blood was then underlaid on 10 mL of Ficoll (Seromed, Berlin, Germany) in 50 mL plastic tube. After centrifugation at 400 × *g* for 20 minutes, PBMC were aspirated from the interface, washed with PBS and resuspended to 4 × 10^6^ cells/mL in complete RPMI 1640 medium [43].

### *In vivo*assay

All mice were maintained in accordance with the NIH Guidelines for the Care and Use of Laboratory Animals with the approval of Review Board of Peking University First Hospital, Beijing. Patient derived xenograft (PDX) model was established as previously described [23]. RCC surgical samples were gently grinded, labeled with Cell Tracker™ CM-Dil dye (MoBiTec, Göttingen, Germany) and subcutaneously injected into NOD/SCID mice (Vitalriver, Beijing, China). The xenograft was then harvested, minced into pieces and transplanted into successive mice. For establishment of RCC cell derived xenograft (CDX) model, 5-week old female BALB/C nude mice (Chinese Academy of Sciences, Shanghai, China) were subcutaneously inoculated with 786-O cells labeled with Dil dye and stably transfected with 3 × 10^6^ pri-let-7d or vehicle control lentivirus. Growth of established xenografts was monitored every two days by a caliper for length (L) and width (W) measurement. Tumor volumes were calculated using the formula (L × W^2^) / 2. *In vivo* treatment of miRNA mimics in PDX model was performed as previously described [23]. 20 nM chemically-modified mi-Ribo™ hsa-let-7d mimics or mi-Ribo™ hsa-let-7d control (Ribobio Co., Guangzhou, China) in 50 μL PBS mixed with 50 μL *in vivo* transfection reagent Entranster™-*in vivo* (Engreen, Beijing, China) were locally injected into the tumor mass once every 3 days for 3 weeks. Quantification of the RCC cell lung metastatic colonies were obtained by examining the mice lung using the TCS 4D laser scanning confocal microscope (Leica, Heidelberg, Germany).

### RNA extraction and real-time RT-PCR

Total RNA was extracted using miRNeasy Mini Kit (Qiagen, Hilden, Germany). For miRNA quantification, 100 ng total RNA was either reverse transcribed directly using stem-loop primers [44], or was polyadenylated with polyA polymerase (NEB, Beverly, MA, USA) then reverse-transcribed with an oligo-dT adapter primer into cDNAs for quantitative real-time PCR [45]. Although both reverse-transcription methods yield reliable and comparative results, the polyA polymerase tailing method was used in this experiments unless specified, given that it allows measuring multiple target miRNAs with one RT reaction. For mRNA analyses, cDNAs were synthesized from 2 μg total RNA, using oligo(dT)_15_ primers and Moloney murine leukemia virus reverse transcriptase (Invitrogen). Quantitative real-time PCR was performed using the SYBR Green PCR Master Mix (Toyobo, Osaka, Japan) in a final volume of 10 μL in ABI 7500 Fast PCR machine. The expression of miRNA and mRNAs were normalized to U6 and GAPDH, respectively. Data are presented as relative quantification (RQ) based on the calculation of 2^-ΔCt^. ΔCt was derived from subtracting the Ct value of reference cDNA from the Ct value of the cDNA of interest. (For a list of all the primers, see Supporting Data, Additional file 2: Table S1)

### Lentiviral transduction

The human pri-let-7d (primary transcript of let-7d) cDNA sequence was synthesized and inserted into the lentiviral shuttle vector plenti6 (Invitrogen) to generate human plenti6-pri-let-7d plasmid. Plenti6 control and human pri-let-7d lentivirus were generated by transfecting 3 μg of plenti6 or plenti6- pri-let-7d and 9 μg of ViraPower Packaging Mix (Invitrogen) into 293FT packaging cells using Lipofectamine 2000. After overnight exposure to the transfection mixture, the medium was changed, and the virus-containing supernatant was harvested 48 h later. The infected cells were selected with 5 μg/mL blasticidin. The antibiotic-resistant clones were pooled and used for subsequent assays.

### *In vitro*cell proliferation, migration, wound healing, and chemotaxis assays

The cell proliferation assay was performed by using Cell Counting Kit-8 (CCK-8, Dojindo, Kumamoto, Japan). Cell migration was evaluated by Boyden chamber assay and wound healing assay [46]. Monocyte chemotaxis was assayed in 24-well transwell plates (Costar #3421) with 5 μm pore polycarbonate filter membrane. Briefly, 1 × 10^7^ RCC cells were cultured in 5 mL complete medium for 24 h, the cultured media were collected after centrifugation and used as conditioned media. PBMC were resuspended in 0.1% BSA-RPMI medium, 4 × 10^5^ cells in 100 μL medium were added to the upper chamber of the 24-transwell apparatus, and 800 μL conditioned medium were added in the lower chamber. After incubation for 8 h, cells that migrated though the membrane were fixed with100% methanol, stained by Giemsa dye, and counted under a microscope. Five high-power fields (×200) were randomly selected and manually counted for each well. The experiment was performed in triplicate (3 wells) with three independent tests. Human recombinant COL3A1 (Fitzgerald, Sudbury, MA) (0.2 μg/mL), human recombinant CCL7 (PeproTech, Rocky Hill, NJ) (10 ng/mL), CCL7 neutralizing antibody and normal goat IgG control antibody (R&D Systems, Minneapolis, MN) (1 μg/mL) were used in these assays.

### Elisa

Cultured media of RCC cells were used for detection of CCL7 by CCL7 ELISA kits (Ray Biotech, Inc). The optical density (OD) at 450 nm was quantified with a Multiskan microplate spectrophotometer (Thermo LabSystems, Milford, MA).

### Quantitative detection of human tumor cell metastasis

The detection of RCC metastasis in mice lung was performed as described previously [47]**.** Genomic DNA was extracted from mouse lung tissues using the EasyPure Genomic DNA Kit (Transgen Biotech, Beijing, China). Quantitative real-time PCR was used to measure human Alu-sequences specific for the most conserved region of humans. The primers for Alu-sequences and PCR conditions were used as previously described [47]. The level of human Alu-sequence was normalized to the amount of mouse/human GAPDH genomic DNA sequence amplified by using mouse/human GAPDH primers [48].

### Immunohistochemistry (IHC) and toluidine blue staining

Paraffin embedded tissues were analyzed using immunohistochemical staining [49] with the following primary antibodies: anti-CD68 antibody (DAKO, Carpinteria, CA), anti-α-SMA antibody (DAKO, Carpinteria, CA), anti-CCL7 antibody (Gen Way Biotech, San Diego, CA), anti-COL3A1 antibody (Bioss, Beijing, China), anti-FOXP3 antibody (Biolegend, San Diego, CA) and rabbit anti-Mouse CD68 antibody (Bioss, Beijing, China). For quantification of tumor stromal cells within the tumor area, CD68 was used as a pan-macrophage marker, α-SMA was used to detect cancer activated fibroblasts adjacent to RCC cells [50], FOXP3 was used as a specific marker for regulatory T cells (Tregs) [22], and mast cells were assessed using the routine toluidine blue staining method [21]. Each tumor section was evaluated by using 20× objective lens, and five independent areas with the most abundant positive cells were selected, digitally photographed, and manually counted under a microscope. The average positive cell counts for each patient were used for statistical analysis. For quantification of CD68^+^ cells in CDX xenografts, four sections from each xenograft were randomly selected and quantified as described above, the average positive cells for each mouse were used for statistical analysis. Results were confirmed by two pathologists in a double-blind analysis.

### Western blot analysis

The lysates were obtained by lysing cells in lysis buffer containing 50 mM Tris, pH7.4, 150 mM NaCl, 0.25% sodium deoxycholate, 1% NP-40, 0.1% SDS, 1 mM PMSF, and complete protease inhibitor cocktail (Roche, Mannheim, Germany). Equal amounts of total protein were subjected to 10% SDS-PAGE and blotted onto PVDF membranes (Pall, Pensacola, FL). Western blotting was performed using rabbit anti-human COL3A1 antibody (Bioss, Beijing, China). The blotting membranes were scanned using GeneSnap acquisition software (Syngene, Cambridge, UK) and band densities were quantified with the GeneTool program (Syngene, Synoptics). GAPDH were used as internal control.

### Dual luciferase activity assay

The 3′-UTR of human COL3A1 and CCL7 containing the putative binding sites and the mutant binding sites of the mature hsa-let-7d were chemically synthesized and inserted immediately downstream of the luciferase cDNA in the pGL3-control vector (Promega, Madison, WI) by GenePharma (Shanghai, China) to form pGL3-COL3A1, pGL3-CCL7 and pGL3-COL3A1-Mut, pGL3-CCL7-Mut constructs. Twenty-four hours before transfection, 786O and 293FT cells were plated at 1.5 × 10^5^ cells/well in 24-well plates. 0.5 μg of pGL3 constructs plus 0.08 μg of pRL-TK (Promega) were transfected in combination with 60 pmol of either a stability-enhanced nontargeting RNA control oligonucleotide or stability-enhanced hsa-let-7d oligonucleotides (GenePharma, Shanghai, China) using Lipofectamine 2000 (Invitrogen). After 48 h, luciferase activity was measured using the Dual Luciferase Reporter Assay System (Promega). Firefly luciferase activity was normalized to renilla luciferase activity for each transfected well. The results were obtained from three independent experiments and each one was performed in triplicate.

### Inhibition of let-7d with a miRNA inhibitor

The chemically-modified mi-Ribo™ hsa-let-7d inhibitor or mi-Ribo™ hsa-let-7d negative control oligonucleotides were synthesized by Ribobio Co. (Guangzhou, China). RCC cells were transfected using Lipofectamine 2000. Cells were collected and assayed at 72 h post-transfection.

### Statistical analysis

Data are presented as mean ± SD and were analyzed using the statistical package SPSS17.0 or GraphPad Prism software 5.0. The significance of differences between two groups was determined using a two-sided Student’s t-test. In case of multiple tests, one-way ANOVA followed by Bonferroni-Holm procedure was applied. Correlation was performed using two-tailed Spearman’s test. *P* ≤ 0.05 was considered statistically significant.

## Electronic supplementary material

Additional file 1: Figure S1: SYBR green real-time RT-PCR analysis shows that there is no significant difference in let-7a expression level between RCC tissue and paired adjacent normal tissue. Horizontal lines represent the relative mean values of let-7a expression for each series of samples. **Figure S2.** SYBR green real-time RT-PCR analysis demonstrates that there is no significant difference in let-7d expression level in adjacent normal tissues between male and female patients. Horizontal lines represent the relative mean values of let-7d expression for each series of samples. **Figure S3.** Representative pictures of positive stromal cells (arrow) in RCC. (Original magnification: ×200). (a) CD68+ macrophages. (b) α-SMA positive cancer associated fibroblasts. (c) Toluidine blue metachromatic mast cells. (d) FOXP3+ T-regulatory cells. (TIFF 4 MB)

Additional file 2: Table S1: Primer sequences. **Table S2.** Statistics of positive cell counts and their correlation with let-7d expression level in tumor tissue. (DOC 56 KB)

## Abbreviations

RCC: Renal cell carcinoma
PDX: Patient derived xenograft
CDX: Cell derived xenograft
Dil: 1,1-dioctadecyl-3,3,3’,3’-tetramethyl-indocarbocyanine perchlorate
RT-PCR: Reverse transcription-polymerase chain reaction
RQ: Relative quantification
ECM: Extracellular matrix.
