# Editorial Expression of Concern: Let-7d suppresses growth, metastasis, and tumor macrophage infiltration in renal cell carcinoma by targeting COL3A1 and CCL7

**DOI:** 10.1186/s12943-023-01778-y

**Published:** 2023-04-26

**Authors:** Boxing Su, Wei Zhao, Bentao Shi, Zhongyuan Zhang, Xi Yu, Feng Xie, Zhongqiang Guo, Xiaoyu Zhang, Jin Liu, Qi Shen, Jinghua Wang, Xuesong Li, Zhiqian Zhang, Liqun Zhou

**Affiliations:** 1 Department of Urology, Peking University First Hospital & the Institute of Urology, Peking University, Beijing, 100034 China; 2 National Urological Cancer Center, Beijing, 100034 China; 3 Department of Cell Biology, Peking University School of Oncology, Beijing Cancer Hospital and Institute, Beijing, 100142 China; 4 Department of Urology, Peking University Shenzhen Hospital, Shenzhen, 518036 Guangdong China; 5 Department of Urological Pathology, Peking University First Hospital & the Institute of Urology, Peking University, Beijing, 100034 China

**Editorial Expression of Concern: Mol Cancer 13, 206 (2014)**

**https://doi.org/10.1186/1476-4598-13-206**

“The Editor-in-Chief is issuing an editorial expression of concern to alert readers that concerns have been raised regarding the images presented in Figs. 2e and 6f [1]. The authors have requested a correction to address these issues, but have been unable to provide suitable data for the corrected image. Readers are therefore alerted to interpret the presented data with caution.

Boxing Xu, Wei Zhao and Liqun Zhou agree to this Editorial Expression of Concern. Bentao Shi, Feng Xie, Zhongqiang Guo, Xuesong Li and Zhiqian Zhang have not responded to any correspondence from the editor or publisher about this Editorial Expression of Concern. The Publisher has not been able to obtain current email addresses for Zhongyuan Zhang, Xi Yu, Xiaoyu Zhang, Jin Liu, Qi Shen and Jinghua Wang.“
