# Supplementary figures and images for: Let-7d suppresses growth, metastasis, and tumor macrophage infiltration in renal cell carcinoma by targeting COL3A1 and CCL7
Source: Mol Cancer. 2014 Sep 6;13:206. doi: 10.1186/1476-4598-13-206 (PMC4168121; doi:10.1186/1476-4598-13-206)

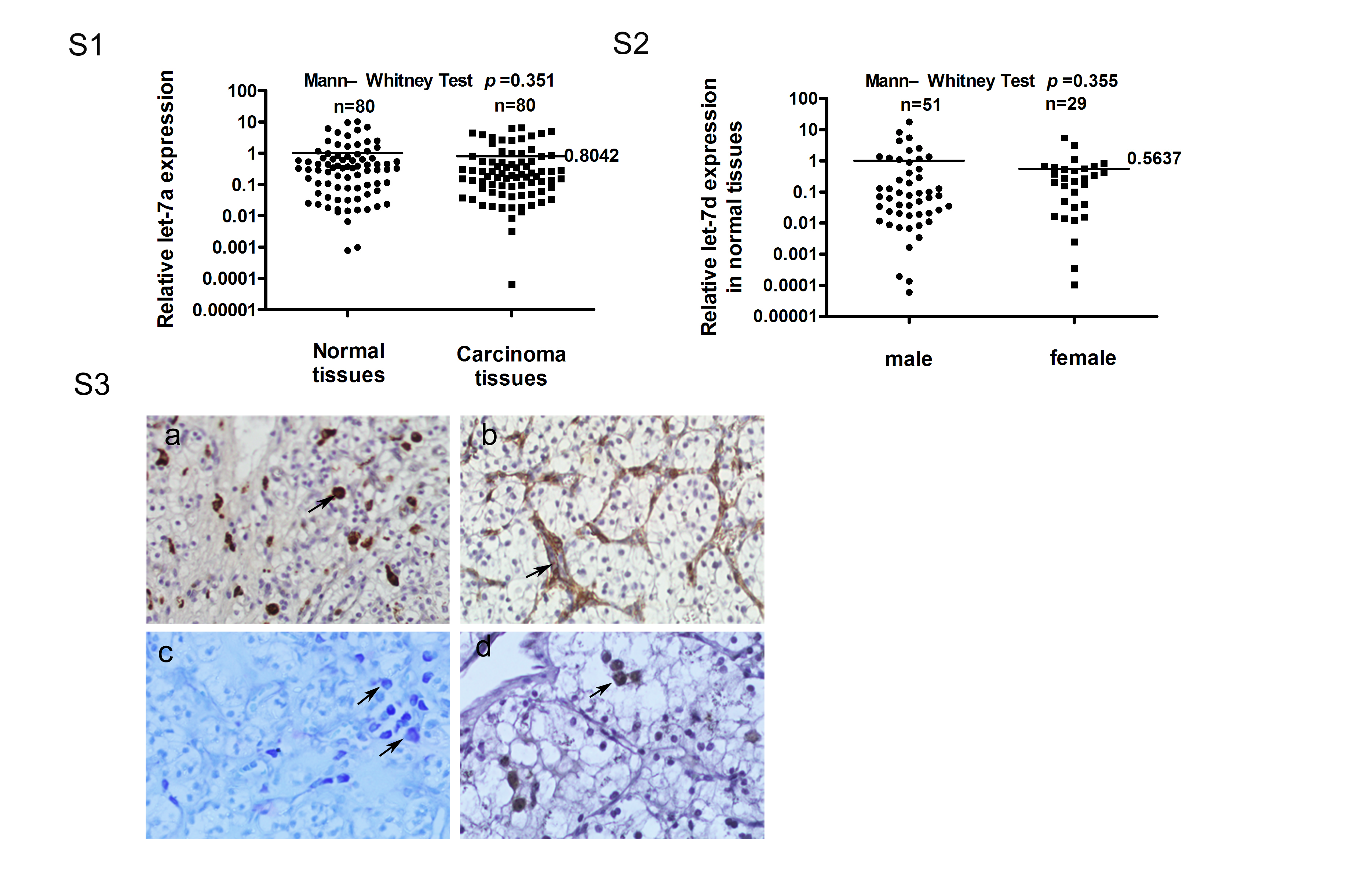

Supplement: Supplementary file 1 — Additional file 1: Figure S1: SYBR green real-time RT-PCR analysis shows that there is no significant difference in let-7a expression level between RCC tissue and paired adjacent normal tissue. Horizontal lines represent the relative mean values of let-7a expression for each series of samples. Figure S2. SYBR green real-time RT-PCR analysis demonstrates that there is no significant difference in let-7d expression level in adjacent normal tissues between male and female patients. Horizontal lines represent the relative mean values of let-7d expression for each series of samples. Figure S3. Representative pictures of positive stromal cells (arrow) in RCC. (Original magnification: ×200). (a) CD68+ macrophages. (b) α-SMA positive cancer associated fibroblasts. (c) Toluidine blue metachromatic mast cells. (d) FOXP3+ T-regulatory cells. (TIFF 4 MB) [file 12943_2014_1406_MOESM1_ESM.tiff]
