# Supplementary material for: Let-7d suppresses growth, metastasis, and tumor macrophage infiltration in renal cell carcinoma by targeting COL3A1 and CCL7
Source: Mol Cancer. 2014 Sep 6;13:206. doi: 10.1186/1476-4598-13-206 (PMC4168121; doi:10.1186/1476-4598-13-206)
Supplement: Supplementary file 2 — Additional file 2: Table S1: Primer sequences. Table S2. Statistics of positive cell counts and their correlation with let-7d expression level in tumor tissue. (DOC 56 KB) [file 12943_2014_1406_MOESM2_ESM.doc]

| **Table S1.** Primer sequences | |
| --- | --- |
| *Alu PCR primers* | |
| Alu forward primer | 5′- ACGCCTGTAATCCCAGCACTT-3′ |
| Alu reverse primer | 5′- TCGCCCAGG CTG GAGTGCA-3′ |
| Mouse/human GAPDH forward primer | 5′- CAGCGACACCCACTCCTCCACCTT-3′ |
| Mouse/human GAPDH reverse primer | 5′- CATGAGGTCCACCACCCTGTTGCT-3′ |
|  |  |
| *PolyA tailing real-time RT-PCR* | |
| oligodT adapter primer | 5′-GCGAGCACAGAATTAATACGACTCACTATAGGTTTTTTTTTTTTVN-3′ |
| Reverse primer | 5′-GCGAGCACAGAATTAATACGAC-3′ |
| Let-7a forward primer | 5′-GGTGAGGTAGTAGGTTGTATAGTT |
| Let-7d forward primer | 5′- TGAGGTAGTTGGTTGTATGGTT |
| U6 forward primer | 5′-AAAATATGGAACGCTTCACGAA-3′ |
| U6 reverse primer | 5′-GTGCTCGCTTCGGCAGCACATAT-3′ |
|  |  |
| *Q-PCR analysis of COL3A1 and CCL7* | |
| COL3A1 forward primer | 5′-GCCAAATATGTGTCTGTGACTCA-3′ |
| COL3A1 reverse primer | 5′-GGGCGAGTAGGAGCAGTTG-3′ |
| GAPDH forward primer | 5′-GACCCCTTCATTGACCTCAAC-3′ |
| GAPDH reverse primer | 5′-CTTCTCCATGGTGGTGAAGA-3′ |
| CCL7 forward primer | 5′-TTGCTCAGCCAGTTGGGATT-3′ |
| CCL7 reverse primer | 5′- GCTCTCCAGCCTCTGCTTAG -3′ |
|  |  |

Table S2 Statistics of positive cell counts and their correlation with let-7d expression level in tumor tissue

| Cells | Mean* | Median* | Variance | Range* | Skewness | Spearman r^#^ | P^#^ |
| --- | --- | --- | --- | --- | --- | --- | --- |
| CD68 positive cells  (macrophage) | 72.46 | 62.72 | 2508.61 | 2.80-208.65 | 0.98 | -0.39 | 0.0003 |
| α-SMA positive cells  (fibroblast) | 40.54 | 43.54 | 657.38 | 0-90.43 | 0.12 | -0.13 | 0.25 |
| FOXP3 positive cells  (Tregs) | 3.57 | 2.75 | 9.98 | 0-11.78 | 0.98 | 0.07 | 0.54 |
| Toluidine blue metachromasia  ( mast cell) | 4.10 | 3.09 | 14.04 | 0-20.09 | 1.32 | 0.15 | 0.09 |

*Data are cells counted per ﬁeld

#Correlations were calculated using Spearman’s r (two-sided).

**Figure S1**

SYBR green real-time RT-PCR analysis shows that there is no significant difference in let-7a expression level between RCC tissue and paired adjacent normal tissue.

Horizontal lines represent the relative mean values of let-7a expression for each series of samples.

**Figure S2**

SYBR green real-time RT-PCR analysis demonstrates that there is no significant difference in let-7d expression level in adjacent normal tissues between male and female patients.

Horizontal lines represent the relative mean values of let-7d expression for each series of samples.

**Figure S3**

Representative pictures of positive stromal cells (arrow) in RCC. (Original magnification: ×200). (a) CD68^+^ macrophages. (b) α-SMA positive cancer associated fibroblasts. (c) Toluidine blue metachromatic mast cells. (d) FOXP3^+^ T-regulatory cells.
